# 

**Published:** 1881-07

**Authors:** 


### CONTENTS.

### COMMUNICATIONS.

Alveolar Ulceration Distinguished from Alveolar Abscess .............. 267
Restoring Partial Loss of the Teeth without Plate.......................... 275
An Ideal .............................................................................. 279
The Fundamental Part of Anatomy—the Bones.............................. 287

CORRESPONDENCE ................................................................ 296

Proceedings of the Iowa State Dental Society................................ 305

### SELECTIONS.

Re-Sharpening Files ............................................................. 306
Tobacco ............................................................................... 306
Disease of the Fifth Nerve....................................................... 307
The Mouth ........................................................................... 307

### EDITORIAL.

In Memory of Dr. James Taylor ............................................... 308
Obituary .............................................................................. 310
International Medical Congress................................................ 311
American Dental Association.................................................. 312
National Dental Association.................................................... 313
Concerning Dental Colleges..................................................... 314

